# A randomised trial of a brace for patellofemoral osteoarthritis targeting knee pain and bone marrow lesions

**DOI:** 10.1136/annrheumdis-2014-206376

**Published:** 2015-01-16

**Authors:** Michael J Callaghan, Matthew J Parkes, Charles E Hutchinson, Andrew D Gait, Laura M Forsythe, Elizabeth J Marjanovic, Mark Lunt, David T Felson

**Affiliations:** 1 Arthritis Research UK Epidemiology Unit, University of Manchester, Manchester, UK; 2 Manchester Academic Health Science Centre, Manchester, UK; 3 Department of Imaging Sciences, University of Manchester, Manchester, UK; 4 Department of Health Sciences, University of Warwick, UK; 5 NIHR Manchester Musculoskeletal Biomedical Research Unit, Manchester Academic Health Science Centre, Manchester, UK; 6 Clinical Epidemiology Unit, Boston University School of Medicine, Boston, Massachusetts, USA

**Keywords:** Knee Osteoarthritis, Treatment, Rehabilitation

## Abstract

**Objective:**

Braces used to treat (PF) osteoarthritis (OA) may reduce contact stress across the PF joint. We hypothesised that in PF OA, braces would decrease knee pain and shrink PF bone marrow lesions (BMLs).

**Methods:**

Eligible subjects had painful PF OA. Subjects were randomly allocated to brace or no brace for 6 weeks. Knee MRIs were acquired at baseline and 6 weeks. We measured BMLs on post-contrast fat suppressed sagittal and proton density weighted axial images. The primary symptom outcome was change in pain at 6 weeks during a preselected painful activity, and the primary structural outcome was BML volume change in the PF joint. Analyses used multiple linear regression.

**Results:**

We randomised 126 subjects aged 40–70 years (mean age 55.5  years; 72 females (57.1%)). Mean nominated visual analogue scale (0–10 cm) pain score at baseline was 6.5 cm. 94 knees (75%) had PF BMLs at baseline. Subjects wore the brace for a mean of 7.4 h/day. 6 subjects withdrew during the trial. After accounting for baseline values, the brace group had lower knee pain than the control group at 6 weeks (difference between groups −1.3 cm, 95% CI −2.0 to −0.7; p<0.001) and reduced PF BML volume (difference −490.6 mm^3^, 95% CI −929.5 to −51.7; p=0.03) but not tibiofemoral volume (difference −53.9 mm^3^, 95% CI −625.9 to 518.2; p=0.85).

**Conclusions:**

A PF brace reduces BML volume in the targeted compartment of the knee, and relieves knee pain.

**Trial registration number:**

UK. ISRCTN50380458.

Symptomatic knee osteoarthritis (OA) affects approximately 12.5% of the US and UK populations age ≥60 years^1^ ^2^ and its prevalence is increasing.^3^ There are few effective non-surgical treatments and none has been consistently shown to affect structural findings in the knee favourably. New effective treatments are badly needed.

The main target of structural modifying trials has been joint space loss on X-ray. Loss occurs slowly and trials testing agents purported to delay that loss require large numbers of patients followed for at least 2 years.^4^ ^5^ Further, knee OA progression is driven by mechanical factors, such as meniscal tears and malalignment across the joint, and drugs that protect against joint space loss may not work in this hostile mechanical environment.^4^ ^6^ For these and other reasons, OA trials targeting structure modification are challenging.

The use of MRI in knee OA studies has revealed structural features that may be treatment targets. Among these are bone marrow lesions (BMLs). Histopathologic studies have shown that these lesions represent areas of microfracture and fibrosis beneath the cortical bone surface^7^ and they have been aetiologically linked to bone trauma. They are caused, in part, by excess focal stress (force per unit area) across a localised area of the joint, such as occurs with malalignment^8^ or a meniscal tear.^9^ Long term studies have shown that these lesions predict adjacent cartilage loss^10^; the development of knee pain correlates with enlargement of these lesions^11^ and a decrease in knee pain is related to their shrinkage.^12^ BMLs wax and wane in size over a period as short as 6 weeks.^13^ Lastly, one recent pilot randomised trial in patients with painful knee OA^14^ showed that zoledronic acid, a bisphosphonate, reduced knee pain and shrank BMLs.

All of this suggests that BMLs may be good structural targets for treatments and that a successful strategy to shrink them might be to reduce focal contact stress across the joint. The knee comprises the tibiofemoral and patellofemoral (PF) joints. PF OA, a common subtype,^15^ is a major cause of pain with stair climbing, arising from a chair, and activities involving kneeling or squatting. Like knee OA in general, treatment of PF OA is limited. One potential treatment is PF bracing. Unlike bulky braces for tibiofemoral OA, these braces are sheer and fit underneath trousers. Powers *et al*^16^ has reported that, by pressing the patella into the trochlear groove, PF braces increase the contact area across the PF joint and thus lessen contact stress; this is because, with the braces on, force becomes distributed across a greater area. To our knowledge, only one trial testing PF bracing has been carried out, a comparative trial testing brace with strap versus brace without strap, and this trial showed no difference between the two conditions.^17^

If PF braces work by decreasing contact stress across the osteoarthritic PF joint, they should, in theory, shrink BMLs which are caused in part by this contact stress. We performed a 6-week randomised controlled trial testing the efficacy of a patellar brace on knee pain and BML volumes as tracked by serial knee MRIs. Because there is no evidence that PF braces affect tibiofemoral joint BMLs which are caused by varus/valgus malalignment, we also hypothesised that tibiofemoral BMLs would not change with PF brace use.

## Methods

### Overview

We carried out a 6-week randomised trial of treatment with a patellar brace for patients with painful PF OA. The primary outcomes were knee pain during a nominated activity and structural change using BML volume in the PF compartment. The trial was carried out from August 2009 through September 2012.

### Subjects

Subjects were recruited from primary and secondary care using letters from general practitioners to knee OA patients, notices in clinics, advertisements in local papers, and referrals from physiotherapists. Subjects were enrolled if their knee radiographs were scored by a musculoskeletal radiologist (CEH) as showing Kellgren and Lawrence grade 2 or 3 in the PF joint (based on either lateral or skyline films), and if this was greater than the grade for the tibiofemoral joint (these grades required at least probable narrowing and definite osteophytes in the PF joint). Subjects were also clinically assessed for PF joint symptoms such as pain with stair climbing, kneeling, prolonged sitting or squatting (we will call these aggravating activities), and on examination by an experienced physiotherapist (MJC) they had to have lateral or medial patellar facet tenderness or a positive patellar compression test. Pain must have been present daily for the previous 3 months and had to be sufficiently severe for a nominated aggravating activity to score 4 or above on a 0–10 cm visual analogue scale (VAS). If both knees were eligible, we asked subjects to select their more symptomatic knee. Potential participants had to be on stable medication for 3 months and were ineligible if they were initiating a new treatment (such as physical therapy). They were asked to remain on the baseline treatment regimen throughout the study and, if randomised to therapy with a brace, were instructed on its use and asked to wear it as many hours during the day as tolerated.

### Exclusion criteria

Subjects were excluded if they had undergone previous patellar surgery. We also excluded subjects with a history of known meniscal or ligament injury, rheumatoid arthritis or other forms of inflammatory arthritis, or an intra-articular steroid injection into the painful knee in the previous month. For the purposes of the MRI, patients were excluded if they had a cochlear implant, metal objects in the body including a joint prosthesis, a cardiac or neural pacemaker, a hydrocephalus shunt, an intrauterine contraceptive device or coil, if they had kidney dysfunction, or were undergoing renal dialysis. Contrast enhanced scans were used in the study to facilitate the quantification of synovial volume. Given the use of these scans, we screened participants for renal dysfunction and excluded those with estimated glomerular filtration rate (eGFR) <45 mL/min. We allowed subjects to enrol even if they did not have PF BMLs at baseline with the anticipation that some would develop these lesions during the trial.

### Randomisation process

Randomisation at a ratio of 1:1 was by pre-prepared sealed opaque envelopes under the supervision of the study statistician.

### Study intervention

Active treatment consisted of a Bioskin Patellar Tracking Q Brace (Ossur UK, Manchester, England; this brace is available throughout the UK and can be seen at http://www.ossur.co.uk/injury-solutions/products/knee/knee-sleeves-wraps/bioskin-q-brace-patella-tracking-brace). At the baseline visit, subjects were randomly allocated to brace or no brace for 6 weeks. The brace has a strap which can be pulled over the patella or it can be worn without the strap. A recent trial^17^ reported no difference in efficacy between the strapped and unstrapped configuration. We allowed patients to select the approach they preferred.

### Outcome measures: pain

The primary symptom outcome measure was knee pain during the pre-specified nominated activity. At the baseline examination, potential participants were asked to select an activity that commonly caused them knee pain and this was checked to make sure it was an activity likely to be related to PF OA. At each visit, subjects completed a 0–10 cm VAS based on the degree of knee pain experienced in the last 7 days during their nominated aggravating activity.

*Knee Osteoarthritis Outcome Score (KOOS)*: The KOOS survey provided secondary pain outcomes. The KOOS is a validated, widely used, knee pain and function survey. We focused on those for pain and function in activities of daily living (ADL) subscales.^18^

### Outcome measures: structure

*PF BML volume:* At baseline assessment, subjects underwent contrast enhanced MRI of their trial knee and then obtained MRIs again using the same protocol and same magnet at 6 weeks. We obtained MRIs with contrast to also assess synovial volume (see below). We studied one knee per person. Using a 1.5 T Philips Gyroscan ACS NT (Philips, Best Netherlands), we obtained axial proton density weighted (PDW) fat saturated (FS) repetition time (TR) 1500 ms, echo time (TE) 15 ms, field of view (FOV) 16 cm, 256×256 matrix, slice thickness 3 mm with 0.3 mm gap, and T1 weighted sagittal post-contrast scans FS TR 500 ms, TE 17 ms, FOV 16 cm, 384×384 matrix, slice thickness 3 mm with 0.3 mm gap in all subjects. The contrast agent was Dotarem (gadoteric acid) at a dose of 0.2 mg/kg. Post-contrast images have been shown^19^ to provide assessments of BMLs similar to PDW FS non-contrast enhanced images.

After initial training from a musculoskeletal radiologist (CEH) on the appearance and size of BMLs and on distinguishing BMLs from non-BML lesions, technicians at iMorphics manually segmented BML volumes in paired images from each subject's knee. BMLs were outlined on each MRI slice and the volume integrated over all slices. During the process of segmentation, the radiologist reviewed any lesions where questions were raised about the nature and size of the lesion, and either he or an experienced radiology trainee (EJM) reviewed all knees showing at least 50% change in BML volume and a random sample of other knees to ensure that these changes were, in reality, changes in BML volumes. As a result of this review, roughly 30% of knees underwent repeat segmentations. For the axial images only, we focused on the patella and femur only. For sagittal images, we segmented BMLs in the patella, femur and tibia. Staff measuring BMLs or other MRI features were blinded to time points and to treatment assignment.

Results were based on the sagittal scan measurements except for the initial 11 patients, who did not obtain these pulse sequences, in which case we used the axial scans (failure to acquire sagittal scans precluded measurement of tibiofemoral BMLs and synovitis in these knees). Interobserver reliability for BML volume was intraclass correlation coefficient (ICC)=0.91 (p<0.001).

**Figure 1:**
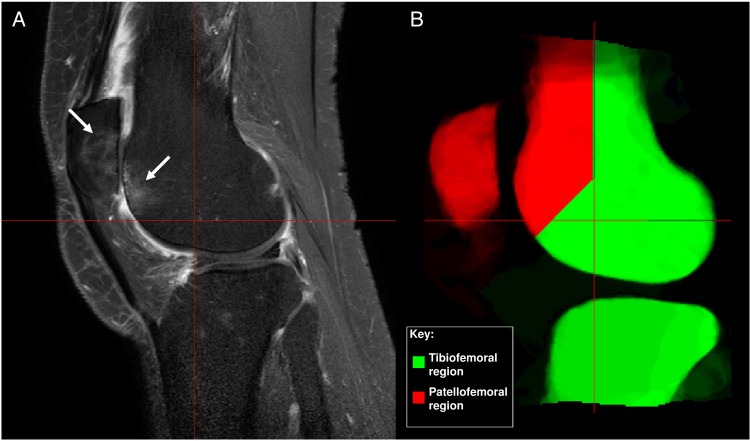
(A) Patellofemoral bone marrow lesions on MRI (arrows). (B) Regions defining patellofemoral and tibiofemoral bone marrow lesions.

The primary structural outcome was change in PF BMLs. We defined BMLs in the PF joint as those involving the patella or the opposing region of the anterior femur using regions derived from the Whole-Organ Magnetic Resonance Imaging Score (WORMS) scale^20^ (figure 1).

We also studied one control structural outcome we hypothesised would not change with treatment, tibiofemoral BML volume, which we determined by taking the whole knee's BML volume and subtracting the PF BML volume.

In addition, we examined a secondary structure outcome, synovial volume. Fluctuation in synovial volume has also been tied to change in knee pain severity,^21^ ^22^ and our contrast-enhanced scans provided us the opportunity to characterise change in synovial volume. Synovial volume was segmented using the same strategy as BML volumes. Reliability of measurement was ICC=0.89 (p<0.001).

### Monitoring adverse events

At the baseline visit, participants were provided with contact information for study staff and asked to call with any problems regarding brace treatment. At the 6-week visit, subjects were asked directly whether they had had any problems or side effects with treatment.

### Sample size

The study was powered to test whether the effects of bracing on pain are mediated by structural changes induced by the brace, using the methods described by Fritz and MacKinnon.^23^ We assumed a moderate effect of bracing on BMLs (an improvement in BML volume of 0.5 SDs in the braced group compared to non-braced) and a moderate effect of BMLs on pain (R^2^ for regression of 0.13). Using a two-sided α of 0.05, and assuming 80% power, 120 subjects would be sufficient to detect the mediated effect.

### Statistical analysis

We used an analysis of covariance (ANCOVA) approach to parallel group trial analysis, to assess between-groups differences.^24^ Six linear regression models were constructed. Each model consisted of one of the outcome variables of interest (eg, pain in nominated activity) at follow-up as the dependent variable, with treatment group as the independent variable and baseline value of the outcome as a covariate. We used an intention-to-treat approach with the last observation carried forward. We also carried out complete case analyses as a sensitivity analysis. Robust standard errors were used, after model residuals suggested evidence of heteroskedasticity. To examine the association of change in pain with change in BML volume, we used linear regression. Statistical analysis was performed using Stata (V.13.1; Stata Corporation, College Station, Texas, USA), with an α level of 0.05 (two-sided) for the assessment of statistical significance.

The study was approved by the Central Manchester Local Research Ethics Committee (Ethics number 09/H1012/35) and Wellcome Trust Clinical Research Facility, Scientific Advisory Board. Subjects provided written consent before randomisation. (UK. ISRCTN50380458.)

## Results

One hundred and twenty-six participants were randomised to brace or no brace. Subjects ranged in age from 40–70 year (mean 55.5 years; SD 7.5). There were 72 females (57.1%). The two groups were similar in terms of demographics and disease measures (table 1). Mean nominated visual analogue scale (0–10 cm) pain score at baseline was 6.5 cm. Participants reported a mean of 7.4 h/day (SD 2.5) of brace use after 6 weeks of treatment. Of the patients who provided data on their patellar strap use, 66% chose not to wear the patellar support strap. Seventy-five per cent of trial knees (94/125) had PF BMLs at baseline.

**Table 1:** Baseline characteristics of patients in the brace trial

Statistic	No Brace Group (N=63) mean (SD)	Brace Group (N=63) mean (SD)
Age	56.4 (8.1)	54.5 (6.7)
% Female	50.8%	63.5%
BMI (kg/m^2^)	30.5 (5.1)	31.4 (6.3)
Baseline pain on nominated activity VAS (0–10 cm)	6.3 (2.1)	6.8 (2.1)
Baseline KOOS pain subscale score (100–0)	51.1 (18.4)	48.2 (18.4)
Baseline KOOS ADL subscale score (100–0)	57.0 (19.2)	52.7 (22.0)
Total bone marrow lesion volume (mm^3^)—all patients	4460.4 (6322.0)	5816.5 (7686.9)
Patellofemoral bone marrow lesion volume—all patients	2088.1 (2938.8)	3039.4 (3974.9)
Tibiofemoral bone marrow lesion volume—all patients	2372.3 (6010.5)	2777.1 (5338.4)
Total bone marrow lesion volume (mm^3^)—patients with BMLs only	4606.7 (6373.0)	6439.7 (7838.9)
Patellofemoral bone marrow lesion volume—patients with BMLs only	2859.9 (3105.4)	3925.9 (4117.3)
Tibiofemoral bone marrow lesion volume—patients with BMLs only	3933.0 (7364.1)	4531.1 (6230.6)
Bone marrow lesion prevalence (N, %):	–	–
Patients with no bone marrow lesions (whole knee)	2, 3.2%	6, 9.7%
Patients with no patellofemoral bone marrow lesions	17, 27%	14, 22.6%
Patients with no tibiofemoral bone marrow lesions	25, 39.7%	24, 38.7%
Total synovitis volume (mm^3^)	29 807.5 (14 469.4)	29 651.5 (13 570.1)
Synovitis prevalence (N, %):	–	–
Patients with no synovitis (whole knee)	0, 0%	0, 0%

All figures are presented as mean (SD) unless otherwise specified.

ADL, activities of daily living; BMI, body mass index; BML, bone marrow lesion; KOOS, Knee Osteoarthritis Outcome Score; VAS, visual analogue scale.

**Figure 2:**
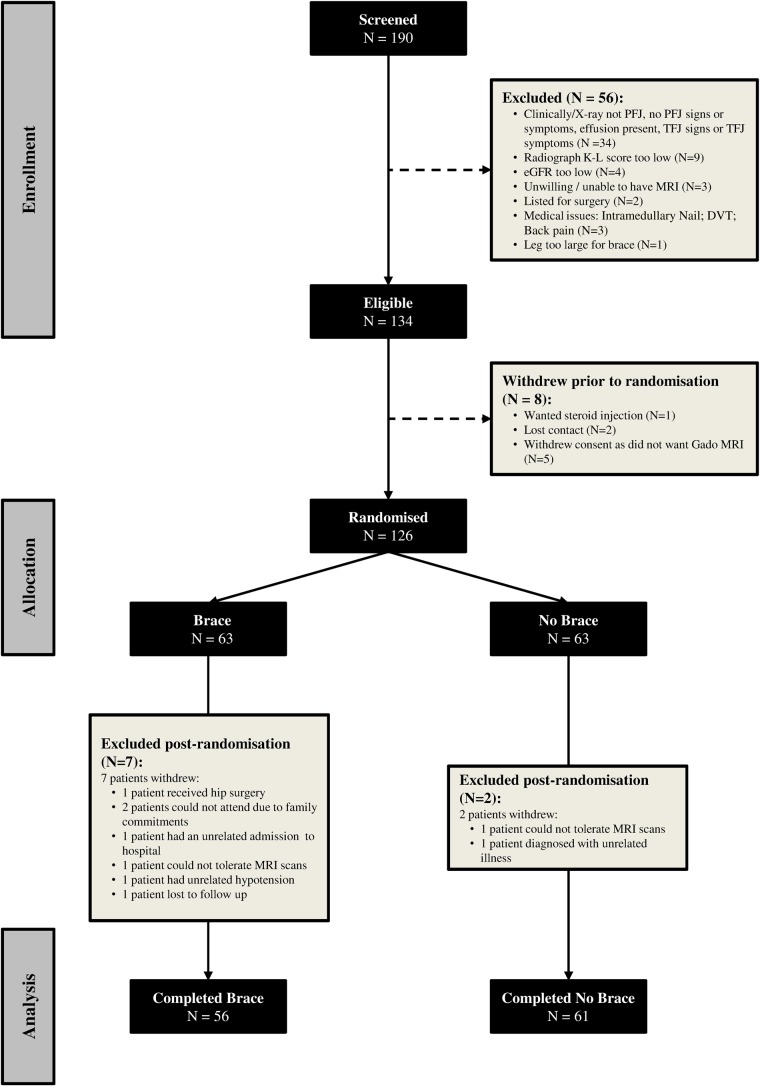
Consort diagram for the brace trial. DVT, deep vein thrombosis; eGFR, estimated glomerular filtration rate; Gado, gadolinium; K-L, Kellgren-Lawrence; PFJ, patellofemoral joint; TFJ, tibiofemoral joint.

Six subjects withdrew during the 6-week trial (4.8%) and were not included in the complete case analysis of pain outcomes (figure 2). One subject did not obtain usable MRI images at baseline and thus 125 participants were randomised in the structural outcomes component of the study. Of these, five were among the six who withdrew and an additional three chose not have to have MRIs again at 6 weeks, precluding analysis of change. Thus, 117 subjects had baseline and 6-week images. One subject had a serious adverse event, bilateral leg swelling, which was felt to be unrelated to treatment (the brace was used on one knee). No other adverse events were reported.

### Pain and symptoms

Compared with the no brace group, the brace group showed a significant improvement in nominated activity pain and in the KOOS subscales (table 2). The results for complete case analysis were similar.

**Table 2:** Results for symptom outcomes: intent to treat analysis (N=125/126/123 for nominated VAS, KOOS pain, and KOOS ADL subscales, respectively)

Variable	No brace	Brace	Between groups difference	
	Mean at follow-up, when controlling for baseline value (95% CI)	Mean at follow-up, when controlling for baseline value (95% CI)	Brace/no brace difference (95% CI)	p Value
Primary outcome: nominated VAS (0–10 cm)	6.3 (5.9 to 6.8)	5.0 (4.5 to 5.5)	−1.3 (−2.0 to −0.7)	<0.001
KOOS pain subscale (100–0)*	51.8 (48.6 to 54.9)	57.5 (53.5 to 61.5)	5.7 (0.6 to 10.8)	0.03
KOOS ADL subscale (100–0)*	56.3 (53.4 to 59.2)	60.8 (58.0 to 63.6)	4.5 (0.5 to 8.5)	0.03

*Increase in KOOS score represents improvement.

ADL, activities of daily living; KOOS, Knee Osteoarthritis Outcome Score; VAS, visual analogue scale.

### Change in structural outcomes

After accounting for baseline values, the brace group had PF BML volumes 490.6 mm^3^ smaller than the group without the brace, representing an 18% smaller volume (table 3). This difference between groups was statistically significant (p=0.03). Similarly, in the complete case analysis, the brace group had PF BML volumes at 6 weeks that were 19.4% smaller than the group not given a brace (p=0.03), after controlling for baseline values. In both intent to treat and complete case analyses, there was a non-significant difference in tibiofemoral BML volumes between the two groups. Lastly, in intent to treat and complete case analyses, synovial volume differed modestly (5–6%) between both treatment groups. Of those who did not have PF BMLs at baseline, three developed BMLs during the trial, and all three were in the no brace group. We found the same trend toward reduction in PF BMLs in those knees where we used sagittal scans and ones where we had only axial scans.

**Table 3:** Results for structural outcomes: intent to treat analysis (N=125 for BMLs and N=106 for synovial tissue)*

Variable	No brace	Brace	Between groups difference	
	Mean at follow-up, when controlling for baseline value (95% CI)	Mean at follow-up, when controlling for baseline value (95% CI)	Brace/no brace difference (95% CI)	p Value
Primary outcome: patellofemoral BML volume (mm^3^)	2709.4 (2426.1 to 2992.8)	2218.8 (1849.7 to 2588.0)	−490.6 (−929.5 to −51.7)	0.03
Control outcome: tibiofemoral BML volume (mm^3^)	2453.1 (2053.9 to 2852.3)	2399.3 (1989.6 to 2808.9)	−53.9 (−625.9 to 518.2)	0.85
Secondary outcome: synovial tissue volume (mm^3^)	29 783.0 (28 517.3 to 31 048.8)	28 096.9 (26 088.4 to 30 105.4)	−1686.1 (−4066.8 to 694.5)	0.16

*This includes knees *w*ith no BMLs in the region at baseline.

BML, bone marrow lesion.

Change in pain on nominated activity was not significantly associated with change in PF BML volume (β coefficient for change in PF BML volume of 1000 mm^3^=0.2 cm, 95% CI −0.1 to 0.4 cm; p=0.17). We also found no significant relation of change in pain with change in overall BML volume, or with change in synovial volume.

## Discussion

In this trial, we found that a PF brace reduced pain in patients with PF OA over a 6-week period. Perhaps more importantly, this brace was associated with a reduced volume of PF BMLs compared with the control. Furthermore, tibiofemoral BML volumes did not differ at follow-up, suggesting that the reduced PF BML volume was specific to the treated knee compartment. Synovial volume change did not differ between treatment groups. We did not find a significant relationship between change in pain and change in any of the MRI parameters.

It has long been thought that much of the pain associated with OA emanates from bone^25^ which is richly innervated with nociceptive fibres. BMLs were noted to be common in knee OA over a decade ago and have been associated with knee pain.^26^ Longitudinal studies have suggested that when these lesions enlarge, pain worsens, and that when they shrink, pain gets better.^12^ Further, these lesions have been linked to subsequent cartilage loss, usually superficial to the lesion.^10^ The histopathology of these lesions suggest they are caused by traumatic focal stress to the bone, and reducing focal stress across the joint should cause them to shrink.

The effect of the brace on pain was modest and the mean difference in pain change versus control was close to the threshold for the minimal clinically important difference for VAS pain, which has varied in different studies.

Bracing has not been widely tested for knee OA. In two of three published trials,^27^ ^28^ valgus bracing for persons with medial knee OA reduced knee pain, although none of these studies examined structural changes. Patellar bracing, which is better tolerated than valgus bracing, has not, to our knowledge, been tested either against placebo or no treatment in a randomised trial. We might have compared patellar bracing against placebo. However, any attempt to enclose the knee in a sleeve or brace could have pushed the patella in and would not have served as an inert placebo. In unpublished work carried out in trial participants using an open MRI, we found that even patellar taping, which can relieve PF pain,^29^ alters patellar position, suggesting that it also would not be an appropriate placebo. We decided therefore to use structural changes to demonstrate effects of the patellar brace.

There was a modest non-signficant association of change in pain with change in PF BMLs. Our failure to find this association is at odds with the other trial using BMLs as an outcome.^14^ Our results, and those of Zhang *et al*,^12^ suggest that the relationship of pain and change in BML volume is modest and would require a much larger sample size than ours.

Our study has several limitations. Most importantly, knee OA is a chronic long term condition. Our 6-week study provides little insight into whether longer term pain or structural deterioration can be affected by this or any other treatment. Work tying these short term to longer term findings is needed. However, it is critical to demonstrate that structural changes can occur quickly in OA as this opens the door to testing many more putative treatments than heretofore feasible. Trials targeting hyaline cartilage protection have had to be large and long term with daunting expense and challenging feasibility, and this has discouraged treatment development. Targeting BMLs may offer an achievable alternative that may expedite and facilitate testing of new treatments.

Our trial focused on a mechanical treatment but, as a recent trial of zoledronic acid^14^ suggests, BMLs may respond to medical treatments. However, our results point to the opportunity that exists for mechanical treatments in a disease where a large component of the pathology is driven by abnormal joint loading.

In conclusion, this trial of bracing for PF OA has suggested that a brace may relieve pain in affected patients. Perhaps more importantly, it suggests that BMLs, a common structural accompaniment of disease, may be treatable.
